# An Examination of Sirolimus's Role in Endothelial Cells of Kaposiform Haemangioendothelioma

**DOI:** 10.1111/jcmm.70903

**Published:** 2025-10-22

**Authors:** Yanan Li, Chuan Wang, Meng Kong, Li Li, Yi Ji

**Affiliations:** ^1^ Department of Pediatric Surgery West China Hospital of Sichuan University Chengdu China; ^2^ Department of Pediatric Surgery Children's Hospital Affiliated to Shandong University Jinan China; ^3^ Department of Pathology, Institute of Clinical Pathology, Key Laboratory of Transplant Engineering and Immunology, NHC West China Hospital of Sichuan University Chengdu China; ^4^ Med‐X Center for Informatics Sichuan University Chengdu China

**Keywords:** angiogenesis, disease model, endothelial cells, Kaposiform haemangioendothelioma

## Abstract

Kaposiform haemangioendothelioma (KHE) research faces challenges due to the lack of established cell lines and suitable animal models. Our study aimed to establish KHE cell lines, spheroids and refine murine models to mimic disease characteristics, advancing our understanding of KHE pathogenesis and exploring novel therapies. Primary KHE cells were sorted using CD31 antibodies and cultured into spheroids. These cells were then injected into mice, and the resulting tumours were analysed using immunohistochemistry. Preliminary exploration of the potential mechanisms of sirolimus action on KHE was conducted through transcriptome sequencing. CD31+ KHE cells were isolated and characterised from three out of six patients. The CD31+ KHE cells demonstrated positive expression of essential markers such as CD31, Ki67 and LYVE1, consistent with the profiles observed in KHE tumours. Additionally, subcutaneous tumours displayed similar positive expression of key markers, reminiscent of KHE tumours. Transcriptome sequencing revealed downregulation of ATG9B after sirolimus treatment in CD31+ KHE cells. CD31+ KHE cells can replicate human KHE in murine models, offering a valuable tool for studying pathogenesis. Our findings also suggest a potential mechanism of sirolimus action in treating KHE, warranting further investigation into novel therapeutic strategies.

## 1 Introduction

Kaposiform haemangioendothelioma (KHE) is a rare vascular tumour, with a reported incidence of 0.9 cases per 100,000 children in the United States between 1991 and 2009 [1]. Initially described in 1993 by Zukerberg et al., KHE is an uncommon soft tissue neoplasm often associated with locally aggressive pathology, consumptive coagulopathy with significant hypofibrinogenemia (Kasabach–Merritt phenomenon, KMP) and lymphangiomatosis [2, 3]. Although KHE is classified as a benign lesion, KMP confers a life‐threatening risk to affected patients. Mortality rates during the period from 1991 to 2009 ranged from 12% to 30%, primarily due to factors such as the compression of vital structures, haemodynamic instability and local invasion [1, 4, 5].

The pathogenesis of KHE involves complex biological processes, and the precise mechanisms driving its development remain incompletely understood [6]. Despite its histological resemblance, the absence of human herpes virus 8 (HHV‐8) in this lesion points to a pathogenesis distinct from that of Kaposi's sarcoma [7]. KHE exhibits unique structural characteristics that promote turbulent blood flow and platelet activation, which account for its association with KMP [8]. While no recurring cytogenetic abnormalities have been consistently identified in KHE, recent findings by Zhou et al. revealed a balanced translocation t(13;16)(q14;p13.3) in a 7‐year‐old male with recurrent KHE [9]. Furthermore, the molecular landscape of KHE remains largely unexplored. KHE research presents several challenges, primarily due to the absence of established tumour cell lines and suitable animal models for in‐depth investigations.

In our previous work, we documented the notable therapeutic effects of sirolimus in treating KHE [10, 11, 12, 13]. However, the absence of readily available KHE cell lines presents a significant barrier to conducting in‐depth mechanistic studies. Consequently, the exact mode of action and specific impact of rapamycin in the context of KHE treatment remains elusive. Further exploration of this topic is crucial to unravelling the intricate dynamics of KHE pathogenesis and devising more targeted and effective therapeutic interventions.

EOMA cells, originating from mouse, are frequently employed in research focusing on haemangioma and KHE [14, 15]. In our previous study, we observed that EOMA xenografts in nude mice closely mimic KHE [16]. Nonetheless, there are notable genetic distinctions between mouse and human cells, leading to varied responses to drugs, diseases and biological processes.

To address these limitations, we have developed novel KHE cell lines, spheroids and refined animal models that closely mimic disease characteristics. These efforts are crucial for advancing our understanding of KHE pathogenesis and exploring novel therapeutic approaches.

## 2 Materials and Methods

### 2.1 Study Design

Following the approval of a human subject protocol by the Committee on Clinical Investigation, KHE specimens were collected from the Department of Paediatric Surgery at West China Hospital of Sichuan University. The clinical diagnosis was confirmed at Sichuan University's West China Hospital's Department of Pathology.

### 2.2 Isolation and Culture of Cells

Single‐cell suspensions were obtained from specimens utilising a previously established collagenase‐based digestion technique [17]. Primary KHE cells were labelled with CD31 antibodies (ab9498, 1:50) for flow sorting of CD31+ KHE cells. Primary KHE CD31+ KHE cells were cultured in endothelial basal medium (EBM‐2, Lonza, Walkersville, MD) supplemented with 1% penicillin–streptomycin solution (HyClone) and 10% foetal bovine serum (Gibco, NY, USA). Cell cultivation was carried out in a humidified atmosphere with 5% CO_2_ at a constant temperature of 37°C. The culture method for EOMA cells is the same as previously reported by us [16].

### 2.3 Rapid Generation of 3D Cell Spheroid

Glass culture dishes with a length‐to‐width ratio of 1.36 were sterilised thoroughly via high‐temperature treatment followed by the application of Sigmacote (Sigma, MO, USA), as we reported previously [16]. These dishes were disinfected before being filled with 10 mL of growth medium, and 3 million cells were seeded within. The dish was shaken at a steady pace of 10 cycles per minute to promote optimal cellular development and interaction. This controlled environment was critical to ensuring a reliable experimental setting. Furthermore, an EVOS TM XL Core microscope (Invitrogen) was used to carefully monitor the microtumour morphology, enabling daily assessment of any observable alterations and developments in the experiment.

### 2.4 Murine Model Mimicking KHE in a Live Setting

Primary KHE cells and CD31+ KHE cells, which were clonally expanded, were suspended in Matrigel and subsequently subcutaneously injected into the right thigh region of 6‐week‐old male nude mice at a concentration of 1 × 10^7^ cells/animal. After 10 days, the animals were euthanised, and the subcutaneous tumours were harvested. These tumours were subsequently fixed in 10% neutral buffered formalin for immunohistochemistry, as described below.

### 2.5 Immunohistochemistry and Multiplex Immunofluorescence

By utilising immunohistochemistry, we conducted a detailed investigation of the distinctive features of the primary KHE cell spheroids, CD31+ KHE cell spheroids, and human KHE tumour tissue. Following fixation with 4% neutral formalin, the samples were segmented and subjected to haematoxylin and eosin (H&E) staining. We selected diagnostic markers of KHE, such as lymphatic vessel endothelial hyaluronan receptor 1 (LYVE‐1) (ab281587, 1:500, Abcam), podoplanin (D240) (ab109059, 1:200, Abcam), platelet endothelial cell adhesion molecule‐1 (CD31) (ab256569 and ab76533, 1:200, Abcam) and haematopoietic progenitor cell antigen (CD34) (ab81289, 1:100, Abcam), for immunohistochemistry and multiplex immunofluorescenc**e**. Ki‐67 (ab16667, 1:100, Abcam) was used to assess cellular proliferation. To evaluate cell adhesion and interactions, N‐cadherin (66219–1‐Ig, 1:100, Proteintech) and E‐cadherin (ab231303, 1:200, Abcam) antibodies were used.

### 2.6 RNA Sequencing

Total RNA was extracted from cells, and its integrity and quality were evaluated. The library products, ranging in size from 200 to 500 base pairs, were subjected to enrichment, quantification and sequencing using Illumina NovaSeq 6000 with the PE150 model following the completion of RNA library preparation and DNA cluster generation.

### 2.7 Quantitative Real‐Time Polymerase Chain Reaction (qRT–PCR)

Total RNA was extracted from cells using TRIzol (15596–026, Invitrogen), followed by cDNA synthesis with an iScript cDNA Synthesis Kit (Bio‐Rad). The endogenous internal control ATCB was utilised. PCRs were conducted in triplicate, and gene expression analysis was performed using Stratagene analysis software and the 2‐ΔΔCt method for quantification. The ATG9B primer sequence was as follows: Forward primer: GGACTCTCCTGGGCTGCGGGTAG, reverse primer: GCAGGCAAAGCCATTCCGCTGGTGG.

### 2.8 RNA‐Seq Data Analysis

The processing and analysis of RNA‐Seq data were performed as in our previous studies [16].

### 2.9 Evaluation of Drug Effects

After 2 days of shaking, the CD31+ KHE spheroid was harvested and seeded into 96‐well culture plates at a density of 1 × 10^4^ cells per well. DNA content was measured in the CD31+ KHE cell spheroid to determine how many cells were present. In addition, the CD31+ KHE cell spheroid was treated with sirolimus (1 nM, 20 nM, 50 nM or 100 nM) for 48 h. The CCK‐8 assay was used to evaluate the viability of the CD31+ KHE cell spheroid exposed to various concentrations of sirolimus (1 nM, 20 nM, 50 nM and 100 nM). The sprouting capability of the CD31+ KHE cell spheroid was examined after transferring those cells into Matrigel 50 μL and treating them with sirolimus (50 nM). After 24 and 48 h of treatment, the CD31+ KHE cell spheroid sprouted and was analysed.

### 2.10 Western Blot Analysis

A Bradford protein assay kit (Bio‐Rad) was used to quantify the protein concentration. Following separation by sodium dodecyl sulphate–polyacrylamide gel electrophoresis (SDS–PAGE), the proteins were electrotransferred onto nitrocellulose membranes. After incubation with primary antibodies at 4°C overnight, the membrane was washed three times and incubated with secondary antibodies. Enhanced ECL‐associated fluorography was used to visualise protein bands. The antibodies used included ATG9B (diluted at 1:1000 for Western blotting) and ACTB/β‐actin (diluted at 1:100000 for Western blotting).

### 2.11 Statistical Analysis

In this study, SPSS 21.0 software (SPSS Inc., Chicago, USA) was used to conduct the statistical analysis. Descriptive statistics, including the mean and standard deviation, are presented for all quantitative variables. Dunnett's test was utilised for quantitative analysis, whereas ANOVA was used for multiple statistical comparisons. Statistical significance was determined at *p* < 0.05, and RNA‐Seq data were analysed using R software version 4.1.0.

## 3 Results

### 3.1 Patient Characteristics

We identified a total of 10 potentially eligible patients with KHE at the Department of Paediatric Surgery, West China Hospital of Sichuan University. Ultimately, six patients provided consent to participate, and skin biopsies were conducted accordingly (Table 1). Subsequently, three strains of CD31+ KHE cells were successfully isolated from these samples.

**TABLE 1:** The clinical characteristics of the patients.

Patient number	Age(m)	Sex	Size(cm)	With KMP	Biopsy site	Successful isolation
1#	6	Female	1.5 × 2	No	Left upper arm	Yes
2#	31	Female	2 × 3	No	Right thigh	No
3#	8	Female	3 × 5	No	Left ankle	Yes
4#	26	Male	3.5 × 4	No	Right calf	No
5#	4	Female	2 × 4	No	Right calf	Yes
6#	38	Male	2 × 3.5	No	Left shoulder	No

### 3.2 Isolation and Characterisation of KHE Cells

**FIGURE 1:**
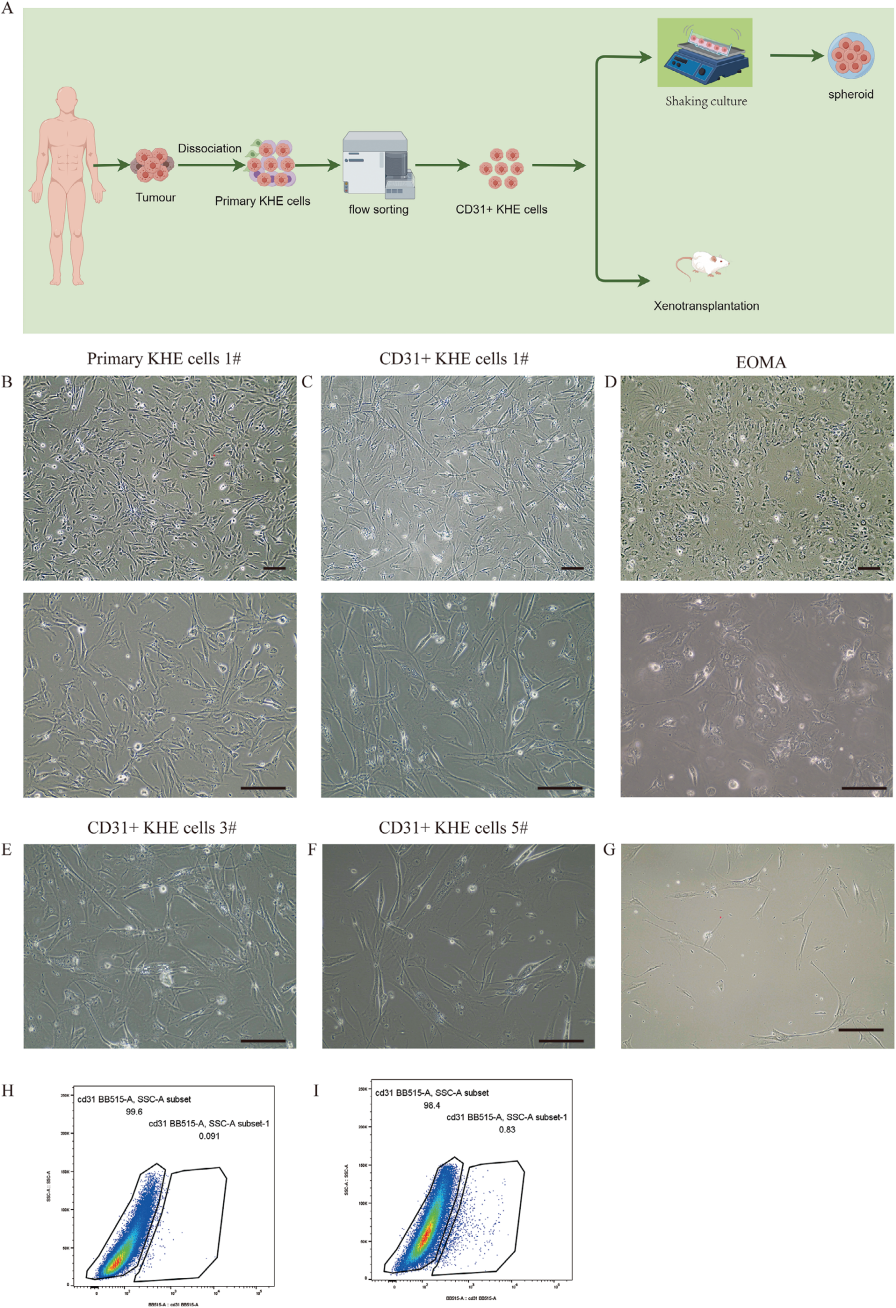
Isolation and culture of KHE cells. (A) The flowchart illustrates the process of sorting CD31+ KHE cells and establishing subcutaneous tumours in nude mice. (B) Morphology of primary KHE cells 1#. (C) Morphology of CD31+ KHE cells. (D) Morphology of EOMA. (E) Morphology of primary KHE cells 2#. (F) Morphology of primary KHE cells 3#. (G) The morphology of CD31+ KHE cells cultured in high‐glucose medium. (H) Flow cytometry sorting results for the control group. (I) The positive results of CD31 flow cytometry sorting. Scale bars = 100 μm.

Primary KHE cells from tumours were isolated, made into suspensions, and then cultured in EBM‐2. Physiologically, the vast majority of primary KHE cells exhibited an elongated morphology, a regular cytoplasmic membrane and small nuclei, as shown in Figure 1B. Using flow cytometry, we isolated CD31+ cells from primary KHE cells and found that approximately 0.83% of the cells were CD31+ cells (Figure 1H,I). We successfully isolated three strains of CD31+ KHE cells from six samples (Figure 1C,E,F). Compared to primary KHE cells, CD31+ KHE cells were larger and more variable in size and shape, as shown in Figure 1B,C. As demonstrated in Figure 1C, the CD31+ KHE cells continued to grow rapidly after passage, expanded further and exhibited a stellate appearance, while Figure 1G shows these cells grew more slowly and unevenly in high‐glucose medium.

### 3.3 Identification and Characterisation of CD31+ KHE Cells

**FIGURE 2:**
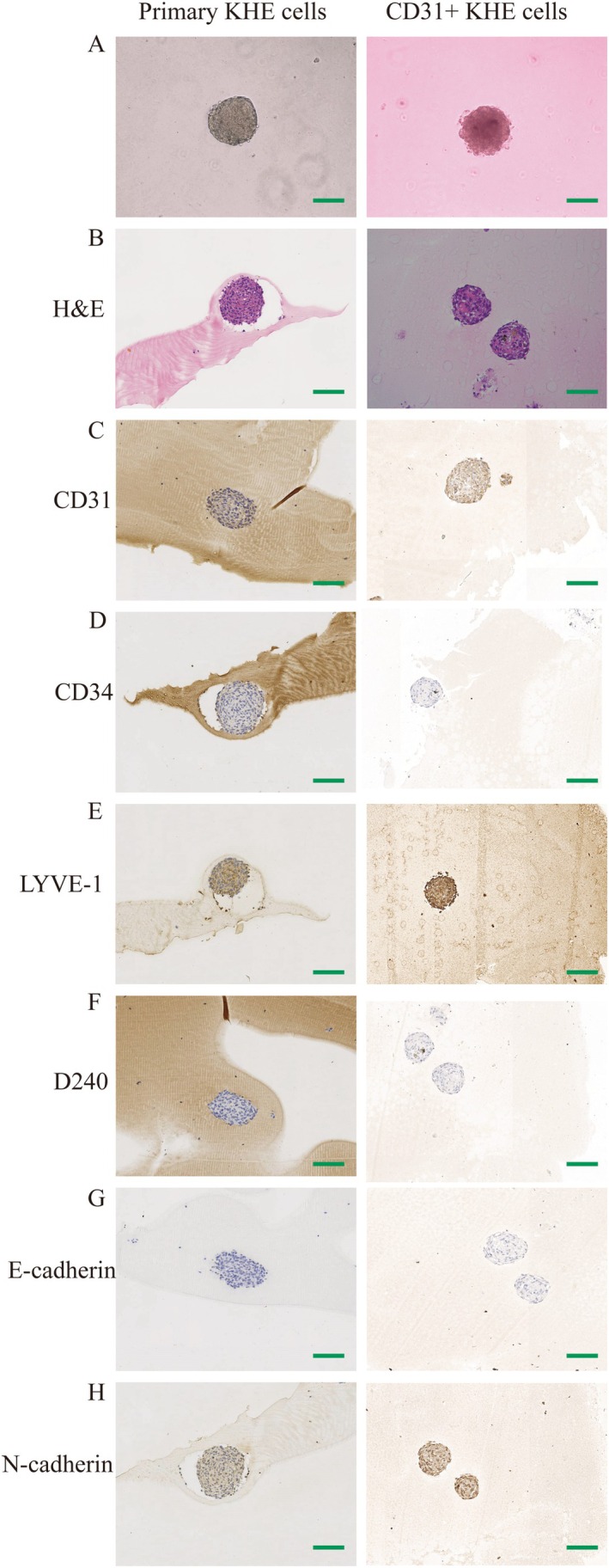
Characterisation of primary KHE cells, CD31+ KHE cells and EOMA. (A) Morphology of primary KHE cells, CD31+ KHE cells and EOMA spheroids. (B) H&E staining of primary KHE cells, CD31+ KHE cells and EOMA spheroids. (C) Immunohistochemical staining of CD31. (D) Immunohistochemical staining of CD34. (E) Immunohistochemical staining of LYVE‐1. (F) Immunohistochemical staining of D240. (G) Immunohistochemical staining of E‐cadherin. (H) Immunohistochemical staining of N‐cadherin. Scale bars = 100 μm.

Primary KHE cells and CD31+ KHE cells were cultured as three‐dimensional (3D) spheroids using a rotary cell culture system to simulate in vivo conditions, as reported by us previously [16]. Compared to the smooth surface of the primary KHE cell spheroid, the CD31+ KHE cell spheroid had irregular shapes with rough surfaces (Figure 2A). Compared to those of primary KHE cells, the nuclei of CD31+ KHE cells were noticeably larger (Figure 2B). Recently, the molecular characteristics of KHE have not yet been elucidated. The diagnosis of KHE is supported by histological characteristics, multiplane involvement and immunoreactivity (100% sensitive, nonspecific) to PROX‐1, LYVE‐1, D240, CD31 or CD34 in neoplastic spindled endothelial cells [8, 18]. Figure 2C reveals that the majority of CD31+ KHE cells stained positive for CD31, in contrast to primary KHE cell spheroids, which were largely CD31‐negative. Interestingly, Figure 2D demonstrates that CD34 staining was absent in both primary and CD31+ KHE cells. Immunofluorescence staining for LYVE1 indicated robust staining in CD31+ KHE cells and weak staining in primary KHE cells (Figure 2E). D240 immunohistochemical staining revealed that all groups were negative (Figure 2F). E‐cadherin, typically expressed in epithelial tissues, is often lost in aggressive cancers, while N‐cadherin, normally present in mesenchymal cells, is overexpressed in some cancers, increasing invasiveness [19, 20, 21]. E‐cadherin immunohistochemistry indicated negative staining in primary KHE cells and CD31+ KHE cells (Figure 2G). Interestingly, the immunohistochemical staining for N‐cadherin demonstrated that all groups were positive (Figure 2H). Compared to that in primary KHE cells, N‐cadherin immunohistochemistry was strongly positive in CD31+ KHE cells (Figure 2H). After CD31‐positive sorting, highly invasive CD31‐positive tumour cells may be obtained from primary KHE cells.

### 3.4 Comparison of the Functional Characteristics of CD31+ KHE Cell Spheroid and CD31+ KHE Cells Through Transcriptome Sequencing

**FIGURE 3:**
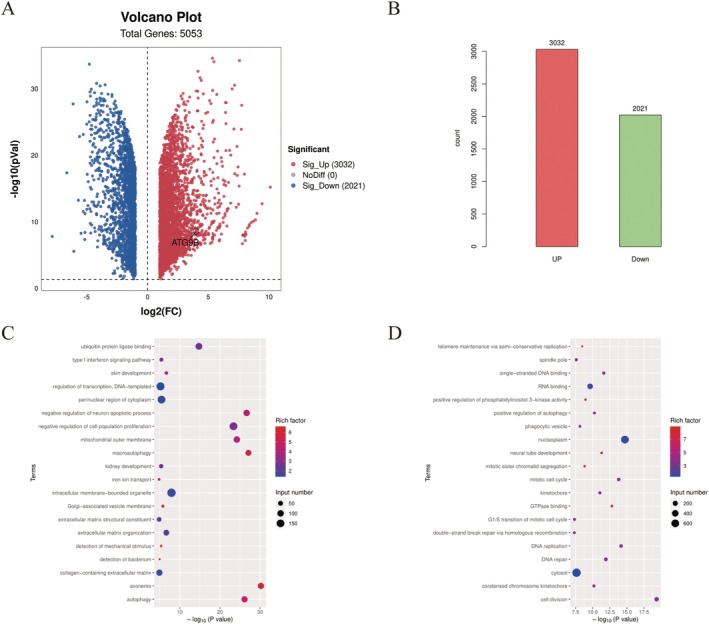
The results of RNA sequencing of CD31+ KHE cell spheroid and CD31+ KHE cells derived from three samples. (A) Volcano plot. (B) Columnar chart of the differential gene numbers. (C) The results of GO enrichment analysis for the upregulated genes. (D) The results of KEGG pathway enrichment analysis for upregulated genes.

Transcriptome sequencing was conducted on CD31+ KHE cell spheroids and CD31+ KHE cells to investigate changes in gene expression during the formation of CD31+ KHE cell spheroids. The analysis identified 3032 significantly upregulated genes and 2021 significantly downregulated genes (Figure 3A,B). Notably, GO enrichment analysis of the upregulated genes revealed enrichment in processes such as autophagy, skin development, macroautophagy, extracellular matrix organisation and collagen‐containing extracellular matrix (Figure 3C). KEGG pathway enrichment analysis of the upregulated genes revealed enrichment of pathways involved in ECM–receptor interactions, autophagy–animal interactions, ferroptosis and complement and coagulation cascades (Figure 3D).

### 3.5 A Xenograft Tumour Formation in Nude Mice

**FIGURE 4:**
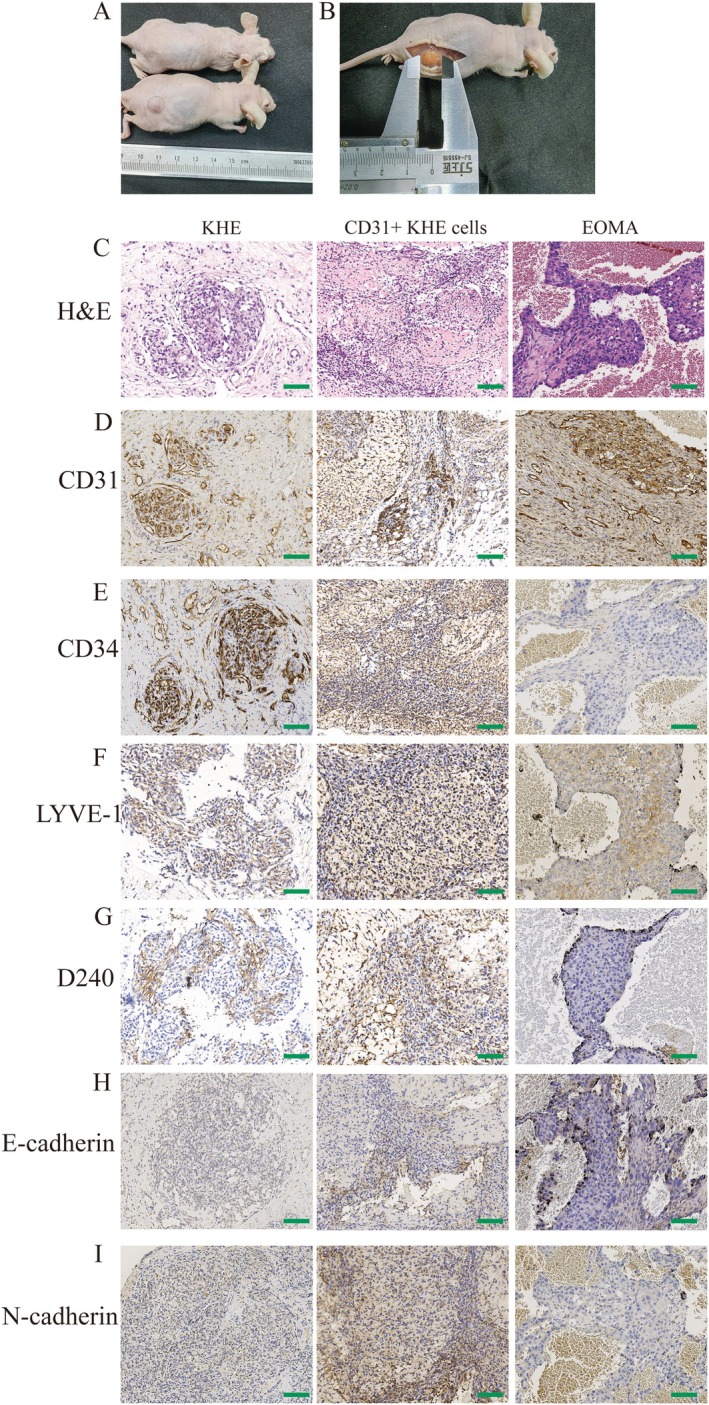
Formation and characterisation of xenograft tumours. **(A)** The formation of xenograft tumours derived from CD31+ KHE cells. (B) The morphology of the xenograft tumour. (C) H&E staining of KHE tumours, subcutaneous tumours derived from CD31+ KHE cells and subcutaneous tumours derived from EOMA. (D) Immunohistochemical staining of CD31. (E) Immunohistochemical staining of CD34. (F) Immunohistochemical staining of LYVE‐1. (G) Immunohistochemical staining of D240. (H) Immunohistochemical staining of E‐cadherin. (I) Immunohistochemical staining of N‐cadherin. Scale bars = 100 μm.

We investigated whether primary KHE cells and CD31+ KHE cells form KHE in nude mice. Clonally expanded primary KHE cells and CD31+ KHE cells were resuspended in Matrigel and injected subcutaneously into nude mice. Ten days after the subcutaneous injection of CD31+ KHE cells, subcutaneous tumours gradually formed in the right thighs of the nude mice. Injection of primary KHE cells did not cause solid tumours in nude mice (Figure 4A). The mean tumour width increased to 9 ± 0.16 mm approximately 10 days after CD31+ KHE cells were subcutaneously injected into the nude mice, as shown in Figure 4B. To better evaluate the efficacy of the models, we generated subcutaneous EOMA cell xenograft tumours in nude mice using a previously reported technique [15]. Subcutaneous tumours were removed from the subcutis of the nude mice, and the tumour tissues were then stained with HE and subjected to immunohistochemistry to assess morphological characteristics. HE staining revealed that the KHE tumour was composed of confluent vascular masses or lobules consisting of spindled, hypercellular endothelial cells (Figure 4C). In CD31+ KHE xenograft subcutaneous tumour tissue (Figure 4C), there were various types of cells, some of which were larger than others. While some cells are sparse, others are crowded closely together. The tissue exhibits different textures and patterns, with some areas forming a reticular or striated appearance and other areas appearing in a nodular or punctate pattern. There are numerous structures that resemble veins. The structural morphology of CD31+ KHE xenograft subcutaneous tumour tissue was comparable to that of KHE tumour tissue. HE staining revealed irregular clusters or nest‐like structures in the EOMA subcutaneous tumour tissue (Figure 4C), as well as bleeding, necrosis and vacuolisation in the surrounding area. Tumour cells have round or oval‐shaped nuclei of various sizes, uneven staining and a large number of mitotic figures. From an overall morphological perspective, there are some differences between EOMA subcutaneous tumour tissue and KHE tissue. The CD31+ KHE subcutaneous tumour cells were positive for CD31, CD34, D240, Ki67, E‐cadherin, N‐cadherin and LYVE1, similar to human tumour cells (Figure 4D–I). However, ICH revealed that the EOMA subcutaneous tumour cells were positive for CD31, CD34, Ki67 and LYVE1 but negative for D240, E‐cadherin and N‐cadherin (Figure 4D–I).

### 3.6 Multiplex Immunofluorescence Results

**FIGURE 5:**
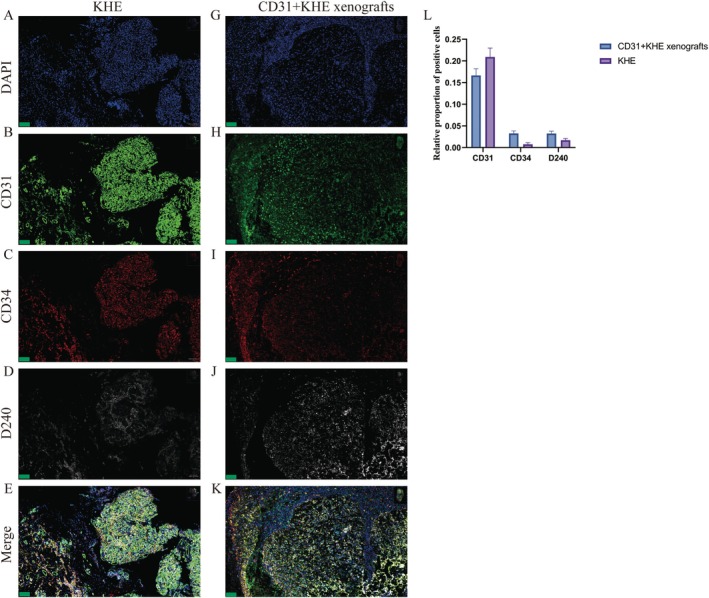
Multiplex immunofluorescence. Features of KHE cells detected by immunocytochemistry with (A) DAPI, (B) CD31, (C) CD34, (D) D2‐40 and (E) merge. KHE features were detected by immunocytochemistry of CD31+ KHE xenografts with (G) DAPI, (H) CD31, (I) CD34, (J) D240 and (K) merge. (L) The relative proportions of CD31‐, CD34‐ and D240‐positive cells among KHE and CD31+ KHE xenografts. Scale bars = 100 μm.

Interestingly, CD31+ KHE cells were negative for CD34, whereas subcutaneous tumour cells (following subcutaneous inoculation with CD31+ KHE cells) were positive for CD34. Multiple immunofluorescence stains were used to examine the distribution of CD31‐, CD34‐ and D240‐positive cells. The findings suggest that the expression patterns of CD31, CD34 and D240 are comparable between KHE and subcutaneous tumours (Figure 5A–K). Nevertheless, the prevalence of CD31 positivity was greater in KHE tumours than in subcutaneous tumours, whereas the rates of CD34 and D240 positivity were markedly greater in subcutaneous tumours than in KHE tumours (Figure 5L). During the subcutaneous tumour formation process, CD31+ KHE cells may attract a significant quantity of CD34‐ and D240‐positive cells.

### 3.7 The Effect of Sirolimus on CD31+ KHE Cell Spheroid

**FIGURE 6:**
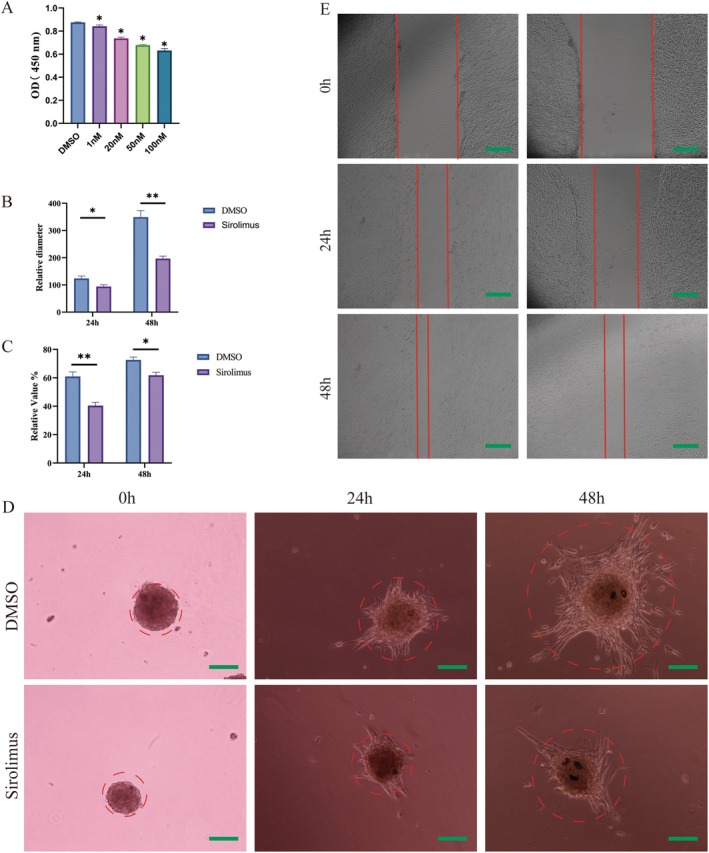
The effect of sirolimus on CD31+ KHE cells and spheroid. (A) Proliferation of CD31+ KHE cell spheroids derived from three samples treated with sirolimus for 24 h. (B) The relative diameter of CD31+ KHE cell spheroid treated with sirolimus for 0, 24 and 48 h. *p < 0.05, compared to DMSO. (C) and (E) The experimental results of the scratch test. (D) The sprouting of CD31+ KHE cell spheroids treated with sirolimus for 0, 24 and 48 h. Scale bars = 100 μm.

Following a 2‐day period of shaking and incubation, CD31+ KHE cell spheroids were collected and exposed to varying concentrations of sirolimus (1 nM, 20 nM, 50 nM or 100 nM). Analysis conducted using the CCK‐8 assay indicated that sirolimus treatment, particularly at concentrations exceeding 20 nM, significantly suppressed cell proliferation in comparison to that in the DMSO control group (Figure 6A). As a result, 50 nM sirolimus was chosen for further examination. Protein was extracted from CD31+ KHE cell spheroids treated with either sirolimus or DMSO for Western blotting. The sprouting of CD31+ KHE cell spheroids was markedly suppressed by 50 nM sirolimus (Figure 6B,D). Additionally, compared with treatment with DMSO, treatment with sirolimus resulted in a notable decrease in the migration of CD31+ KHE cells (Figure 6C,E).

### 3.8 Transcriptome Sequencing of CD31+ KHE Cells Following Sirolimus Intervention

**FIGURE 7:**
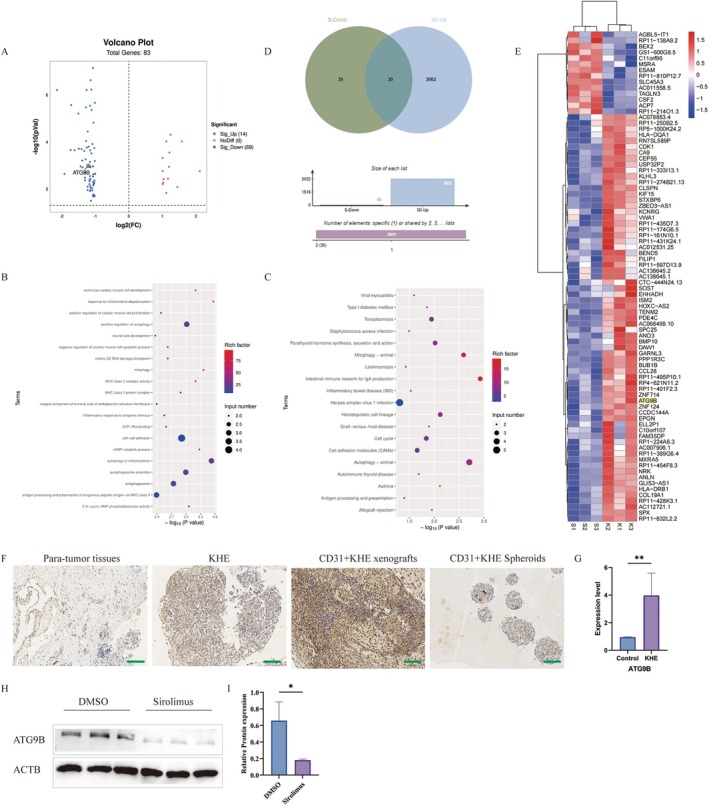
Transcriptome sequencing analysis of CD31+ Kaposiform haemangioendothelioma (KHE) cell spheroid derived from three samples after sirolimus treatment. (A) Volcano plot. High relative expression is indicated by red, whereas low relative expression is indicated by blue. (B) The results of GO enrichment analysis for downregulated genes. (C) The results of KEGG pathway enrichment analysis for downregulated genes. (D) Venn diagram of genes downregulated after sirolimus treatment that intersected with genes upregulated during cell sphere formation. (E) Heatmap of the results. (F) Immunohistochemical staining of ATG9B in para‐tumour tissues, KHE tissues, subcutaneous tumours and CD31+ KHE cell spheroid. (G) Gene expression levels of ATG9B. *p < 0.05, KHE tissues compared to para‐tumour tissues. (H) Western blot showing the expression levels of ATG9B in CD31+ KHE cell spheroids treated with DMSO and sirolimus. (I) Semiquantitative WB analysis. Scale bars = 100 μm.

RNA sequencing was performed on CD31+ KHE and CD31+ KHE cell spheroids (treated with sirolimus) to examine alterations in gene expression following sirolimus treatment. The analysis revealed 14 significantly upregulated genes and 69 significantly downregulated genes (Figure 7A). Significantly, the GO enrichment analysis of the downregulated genes indicated enrichment in various processes, including positive regulation of autophagy, mitophagy, MHC class II receptor activity, cell–cell adhesion and autophagy of mitochondria (Figure 7B). Furthermore, KEGG pathway enrichment analysis of the downregulated genes revealed enrichment of pathways such as mitophagy–animal, autophagy–animal, haematopoietic cell lineage and cell adhesion molecules (Figure 7C).

After sirolimus treatment, a total of 30 related genes (downregulated genes after sirolimus treatment intersected with upregulated genes during cell sphere formation), including the autophagy‐related gene ATG9B, were upregulated during CD31+ KHE cell sphere formation (Figure 7D,E). ATG9B was expressed more highly in CD31+ KHE cell spheroids, subcutaneous tumours and KHE tissues than in para‐tumour tissues (Figure 7F). qRT–PCR confirmed that ATG9B was substantially upregulated in KHE (Figure 67G). The expression of ATG9B was significantly lower in the 50 nM sirolimus group than in the DMSO group (Figure 7H,I). These findings indicate that ATG9B may play a crucial role in the development of KHE and that the proliferation of CD31+ KHE cells may be suppressed by sirolimus through the downregulation of ATG9B expression.

## 4 Discussion

In this study, we successfully isolated CD31+ cells from primary KHE cells, representing a critical step forward in KHE research. These cells were morphologically similar to those of primary KHE. Remarkably, the CD31+ KHE cells exhibited positive expression of key markers, including CD31, Ki67 and LYVE1, mirroring the profiles observed in KHE tumours. More importantly, in this research, we demonstrated a significant breakthrough by utilising CD31‐selected KHE cells to replicate human KHE in an in vivo murine model. Although CD34 and D240 staining are negative in CD31+ KHE cells, the subcutaneous tumour cells derived from the CD31+ KHE population exhibited positive immunoreactivity for several key markers, including CD31, CD34, D240, Ki67 and LYVE1.

In our prior studies, we utilised CD31 as a key marker to distinguish and characterise tumour‐initiating cells derived from infantile haemangioma [17, 22]. This approach has enabled us to gain significant insights into the cellular properties and behaviour of these specific cells. By utilising CD31, we not only identified these tumour‐initiating cells but also explored their potential role in the progression of infantile haemangioma [23]. These findings pave the way for a deeper understanding of the disease and have important implications for potential therapeutic strategies. Kai Li's earlier work involved the isolation of primary cells from KHE, yet their attempts to establish a murine model through the direct injection of these primary KHE cells proved unsuccessful [24]. In our study, we adopted a different strategy by isolating CD31+ cells from primary KHE cells. Through the precise flow cytometry process, we successfully isolated approximately 0.83% of the cells. Of particular interest is the remarkable behaviour of these CD31+ cells when cultured in vitro. These cells exhibited a rapid proliferation rate and self‐renewal ability. Subsequently, when we introduced these CD31+ KHE cells via subcutaneous injection into nude mice, we observed the gradual formation of subcutaneous tumours in the right thigh area within 7–10 days.

EOMA cells, a murine‐derived cell line, are frequently utilised in haemangioma and KHE research [14, 15]. Nonetheless, considerable genetic disparities are evident when comparing mouse cells to human cells [25]. These distinctions involve variances in chromosome count, chromosome structure, genome composition and gene expression profiles [26]. As a result, mouse cells and human cells display noteworthy genetic differences, which can result in diverse reactions to various drugs, diseases and biological processes. This difference may also be one of the factors limiting KHE research. ICH analysis revealed a distinct pattern in the cells of the EOMA subcutaneous tumours. These cells exhibited positive staining for CD31, CD34, Ki67 and LYVE1, reflecting their specific cellular characteristics. However, they were found to be negative for D240, with a slight variance compared to KHE tissue. The subcutaneous tumour cells derived from the CD31+ KHE population exhibited remarkable positivity for an array of key markers. Specifically, these cells strongly expressed CD31, emphasising their endothelial nature. Furthermore, the Ki67 marker, which signifies proliferative activity, was prominently expressed in these cells. This observation suggested that CD31+ KHE subcutaneous tumour cells possess a high degree of cellular proliferation, which is often associated with KHE. Notably, the presence of LYVE1 implies lymphatic endothelial differentiation, further validating its resemblance to human KHE cells. In CD31+ KHE cells, CD34 and D240 were negative, whereas in the subcutaneous tumours of nude mice, CD34 and D240 were positive. Through multiplex immunofluorescence staining of CD31, CD34, D240 and LYVE1 in KHE tissues and subcutaneous tumour tissues of nude mice, we found that they exhibited similar distributions of positive cells. We speculate that during the process of subcutaneous tumour formation in nude mice, CD31+ KHE cells may recruit CD34‐ and D240‐positive cells to participate in tumorigenesis. This finding reveals insights into cellular interactions during tumour formation, providing new clues and directions for KHE research. This comprehensive marker expression profile not only supports the validity of CD31^+^ KHE cells as a representative model but also provides valuable insight into their cellular properties.

At present, the Food and Drug Administration (FDA) in the United States has not approved any pharmaceutical treatments for KHE [11]. Research conducted since 2010 has demonstrated the distinctive therapeutic efficacy of sirolimus for treating KHE [27, 28, 29]. The results of our clinical trial indicate that the combined administration of sirolimus and prednisolone is highly efficacious in the treatment of KHE with KMP [11]. However, the specific mechanism through which sirolimus improves KHE is still not fully understood. Previous research conducted by our team demonstrated a substantial decrease in EOMA cell proliferation when cells were treated with sirolimus at a concentration of 50 nM [16]. Similarly, in the present study, we observed marked suppression of CD31^+^ KHE cell proliferation at the same concentration. In the present study, following sirolimus treatment, we identified a group of 30 genes that were upregulated during the development of CD31+ KHE cell spheroids, among which was the autophagy‐related gene ATG9B. Recent studies have revealed the potential of autophagy inhibition as a viable approach to attenuate the aggressiveness of cancer stem cells, thereby enhancing their susceptibility to therapeutic interventions. The dualistic nature of autophagy in regulating stemness traits presents a complex challenge due to its divergent impacts on the progression of cancer [30, 31]. Autophagy plays a crucial role in facilitating the self‐renewal and therapeutic resistance of cancer stem cells, whereas autophagy deficiency impedes the differentiation of stem cells and enhances tumorigenic potential [32]. Research by Xiu‐Wu Bian has highlighted the essential role of ATG9B in both self‐renewal capacity and tumour propagation potential [30]. The findings of their research suggested that the interplay between stem cell properties and autophagy, regulated by the ASCL2‐ATG9B pathway, significantly influences the progression of gliomas. Importantly, the ASCL2‐ATG9B pathway may serve as a promising biomarker for prognosis and therapeutic intervention. Our study revealed a noteworthy decrease in the expression of ATG9B in the 50 nM sirolimus‐treated group compared to the DMSO control group. These results indicate that sirolimus may inhibit the proliferation of CD31+ KHE cells by suppressing the expression of ATG9B. In future studies, we will further explore the role and mechanisms of ATG9B in the development and progression of KHE.

This study has several limitations, including a relatively small overall sample size in which only three primary cell lines were successfully isolated from six samples. Additionally, only one primary cell line demonstrated tumorigenic potential when implanted subcutaneously in nude mice, and no KMP events were observed. Future studies would benefit from larger sample sizes to more comprehensively investigate these findings.

At present, the pathogenesis of KHE is not well understood, and the specific mechanisms by which sirolimus affects the treatment of KHE remain unclear. In this study, we successfully established CD31+ KHE cells, spheroids and animal models. The generation of CD31+ KHE cells may allow us to delve deeper into the complicated cellular mechanisms involved, allowing for a more comprehensive understanding of its pathophysiology and the possibility for therapeutic approaches in the future.

## Author Contributions

**Yanan Li:** writing – original draft (equal). **Chuan Wang:** writing – original draft (equal). **Meng Kong:** writing – original draft (equal). **Li Li:** writing – original draft (equal). **Yi Ji:** writing – original draft (equal).

## Ethics Statement

Informed consent was obtained from the patients in accordance with the principles of the Helsinki Declaration. The Ethics Committee of West China Hospital approved this study (Approval No: 2019 (1085)). The IACUC committee and the Animal Experiment Center at Sichuan University approved all the experimental protocols (Approval No: 20220307040).

## Consent

The authors have nothing to report.

## Conflicts of Interest

The authors declare no conflicts of interest.

## Data Availability

The datasets used during the current study are available from the corresponding author on reasonable request.
